# Ten Simple Rules for Making a Career Transition from Basic Science to Public Health Research

**DOI:** 10.3390/ijerph22020223

**Published:** 2025-02-05

**Authors:** David Berrigan, David M. Hartley

**Affiliations:** 1 Health Behaviors Research Branch, Division of Cancer Control and Population Sciences, National Cancer Institute, Rockville, MD 20853, USA; 2 Cincinnati Children’s Hospital, James M. Anderson Center for Health Systems Excellence, Cincinnati, OH 45229, USA; david.hartley@cchmc.org; 3 Department of Pediatrics, College of Medicine, University of Cincinnati, Cincinnati, OH 45221, USA

**Keywords:** career change, basic science, public health, research, training

## Abstract

It is not uncommon for basic scientists to switch into public health research. Such career transitions present a variety of challenges and opportunities and can reinvigorate a career, lead to new skills, and provide the chance to contribute to individual and community health and social justice. Based on our respective experiences switching from applied physics to infectious disease modelling and from evolutionary physiology to cancer prevention and control, we propose ten simple rules intended to help researchers from other disciplines think about a transition to public health research. Together, these rules are largely about navigating between pairs of extremes related to why you want to move in a new direction, how to balance old and new expertise, and balancing humility with the confidence that you are bringing something important to the table. A career transition can also fulfill some of the basic motivators for a research career, including curiosity and a passion to try to solve important problems. Our career transitions proved deeply satisfying. We hope yours will as well.

## 1. Introduction

It is not uncommon for basic scientists to switch into public health research. We, David B. (D.B.) and David H. (D.H.), neighbors in Montgomery County, Maryland, have often talked about our career trajectories. For DB, this included a move from insect evolutionary physiology into cancer prevention and control, and for DH, this included a move from applied physics to the mathematical modelling of infectious diseases. A career transition such as the ones we made can be energizing and transformative, but it can also be daunting. While switching research areas is no guarantee of creative new contributions to science, we do think teams with diverse disciplinary backgrounds can be more fun, more creative, and sometimes more productive. On the other hand, great care must be taken to remember differences in training and vocabulary and the blind spots associated with coming to a new area of research [1].

We hope these ten rules will supply some things to think about if you are considering such a move rather than a roadmap of exactly how to make such a transition. Based on our own experiences, we emphasize a transition at the doctoral level, but many of these thoughts could apply to transitions prior to obtaining a PhD.

## 2. Ten Simple Rules

**Rule 1: Have a strong motivation to leave your current research area**

We think of this as “The Push”. It is not easy to transform your research career in a dramatically new direction. This makes it important to have a strong motivation to make such a transition. For example, academic positions in evolutionary biology almost uniformly require significant teaching. When DB was a postdoctoral student, he taught a course for his advisor during his sabbatical and realized he did not enjoy lecturing for a whole course. This strongly motivated him to consider new directions. Funding can also provide a strong motivation to look for a new direction. For DH, a National Research Council physics postdoctoral fellowship went unfunded due to a federal budget error and thus a job opportunity in industry that involved working in disease modelling and public health was welcome. Of course, any research career will require time spent on activities that are not your favorite, so think carefully as to whether you are really motivated to switch or are just thinking that the “grass might be greener on the other side of the hill”.

**Rule 2: Have a good reason for entering your chosen aspect of public health**

We think of this as “The pull”. A move into public health research can appeal for a variety of reasons, i.e., ‘pulls’. For example, when DB and his wife started a family, he felt a strong impetus to carry out research with a more proximal effect on human wellbeing. Basic research on insect evolutionary responses to temperature seemed relevant to climate change science, but an awfully long way from influencing its trajectory. His switch into cancer prevention research offered the prospects of work more closely connected to health and health equity. Furthermore, research in public health is based on a perspective that there are multilevel influences on health and that prevention is an important goal for health research. These and other factors can motivate a switch from what may seem like a very technical and focused research question in the basic sciences.

You may find the new field and research more interesting than your home field. Other personal experiences can be motivating as well, including the career trajectories of friends and family, experiences of illness, and engagement with the health care system. Lastly, for many people, simply learning about new areas of science can be rewarding and attractive, addressing curiosity, a strong motivator for many researchers [2].

**Rule 3: Bring something to the table**

The fact that you want to transition into public health research may not alone motivate people to give you opportunities. For DH, broad knowledge of mathematics and programming allowed him to begin reading the computational and mathematical epidemiology literature and working with models. Growing awareness of emerging infectious disease and other world events provided opportunities to apply modelling and mathematical skills. For DB, a strong background in physiology, genetics, and population ecology was valued by a training program aimed at bringing researchers with diverse backgrounds into cancer prevention and control [3]. Some evidence suggests that cross-disciplinary teams have increased scientific productivity [4]. This supports the value of bringing researchers from diverse disciplines into public health research.

**Rule 4: Embrace new ways of thinking**

Public health research covers a lot of ground, and some of its elements will challenge you. Public health blends diverse disciplines and emphasizes social justice, causal inference, health psychology, exposure assessment, clinical practice for prevention, and many other facets of promoting and protecting the health of all people and their communities. Many activities of public health research will be familiar to basic scientists, such as hypothesis generation and testing, data collection and analysis, and dissemination of findings. On the other hand, basic scientists will be surprised by the challenge of causal inference when experimental studies are impractical or unethical. This will not be true for all disciplines. For DH, inference in surface physics often depends on observational results, and similarly for DB, evolutionary biology, like epidemiology, involves inference about past exposures and their consequences. An attraction and possibly a challenge will also be the public health emphasis on social justice. DB vividly remembers when the professor (Dr. Lawrence Wallack) of his first MPH course at UC Berkely strode into the classroom and wrote “Public Health is Social Justice”. This blew his mind after a decade counting and sexing *Drosophila* under the dissecting microscope and has stuck with him as a powerful motivator for continued efforts in public health research.

**Rule 5: Take the time and effort to learn the new field**

With a PhD and experience in another field, you may think you already know how to conduct research (Rule 7), but public health research is complex (Rule 4). Spending adequate time understanding the discipline you are entering, its history, and its intellectual approaches is vital if you are going to succeed and make some genuine contributions in your new area.

Consider an additional degree. Many schools have one-year master’s in public health (MPH) programs for people with advanced degrees in other disciplines. Both DB and DH completed MPH programs. For DH, the MPH helped him to appreciate that biological and sociological sciences are fundamentally different from physics and completely valid. For DB, the MPH opened his eyes to the problem of causal inference and the intense focus of epidemiological research on this challenge. This has proved invaluable in his efforts to help promote NIH funding for observational studies addressing natural experiments [5,6].

But remember, a further degree is good but not necessary. It can be good because it fills in some holes, inculcates you with new ways of thinking, teaches you the language of the new field, and confers a recognizable credential. However, the cost in time and money may be considerable, and self-study and “learning on the job” work for many. Of course, these approaches require excellent mentors and self-discipline.

**Rule 6: Do not forget or reject your past work and identity**

Some of the reasons for changing careers can be complicated. Examples might include loss of funding, difficult work environments, and feelings of failure or indifference related to a past area of research training and effort. This can lead to a desire to ‘shut the door’ on the past, but melding your past experiences, training, and perspectives with your new direction is highly valuable. It can help you to bring something new to the table, and it is psychologically beneficial to forge this new identity and add renewed meaning to your work [7]. Some of the career changing researchers in the Cancer Prevention Fellowship Program think of this as ‘building a bridge’ between their old and new research directions rather than just jumping across to the new terrain.

**Rule 7: Adapt and sell your high-level modelling and statistical skills**

There is a perennial and strong demand for quantitative and modeling skills all across public health research. The continuing ubiquity of “big data”, linkage studies, geospatial approaches, systems modelling, and other analytical approaches are only reinforcing this demand [8,9,10]. DH was able to apply math skills to epidemic modelling and public health surveillance after his field switch, and when COVID-19 emerged, he found himself deeply involved in developing situational awareness tools in support of a regional response [11,12,13]. For DB, quantitative genetics training, evolutionary optimization modelling of the evolution of age and size at maturity, and statistical skills made him an attractive fellowship candidate even though he did not end up using these genetic and mathematical approaches very directly in his subsequent work. If you lack such skills, brushing up your analytical and statistical toolkit will be very helpful in a large part of contemporary public health research. Common statistical software packages used by public health researchers include SPSS, SAS, and Stata, with R also growing in popularity in recent years [14].

**Rule 8: Talking to people is part of your public health research**

For many basic scientists, human behavior, attitudes, and cognitions have little or nothing to do with their research. However, these factors are inextricably intertwined with all aspects of public health research. DB recalls a well-known ecologist advising him to spend the first months of his PhD just walking around in different habitats. This perspective has been confirmed for him by readings in sociology calling for efforts to immerse yourself in a context before beginning quantitative work [15,16]. Qualitative work including key informant interviews, focus groups, cognitive interviewing, and other related approaches can go a long way towards better understanding public health relevant attitudes and cognitions. Similarly, DH has found himself involved in qualitative (structured interviews) and mixed-methods research and has found it interesting and valuable. In the midst of his PhD program in physics, however, he would certainly have scoffed at such approaches.

**Rule 9: Be humble about your new research directions**

Depending on the trajectory of your career transition, you may feel at times that you are already an accomplished scientist and have a lot to offer. That may be true but remember there will be a lot you do not know, and it may take years to achieve your ‘black belt’ in public health. You will also continue to note holes in your training long after that. People learn differently at different ages and learning while completing a PhD is very different from learning in the context of a new career, during part time study, and when life is much busier later in a career. These differences have plusses and minuses. Time can be a profound pressure but you also may be more efficient and targeted in fulfilling needs for new knowledge and skills.

A proper measure of humility will also endear you to your new colleagues and community, a huge plus as you seek their collaboration, guidance, mentorship, and support in this new step in your scientific career.

**Rule 10: Be proud of your new research directions**

Scientific training can be intense and can create a powerful sense of identity. This can make change a challenge. For some years, DB lamented that he had, in a sense, ‘given up’ by switching into a new research area and a Federal Government setting, instead of becoming a professor of biology like his mentors. Gradually, he came to realize there are many good ways to be a scientist, not just following in the footsteps of his PhD advisor.

For DH, his transition and worldview started out somewhat differently. After he found infectious disease epidemiology and realized that his physics education could be applied to work on infection, he never looked back. While he never considered himself to be at the forefront of his new field, he simply had fun learning about everything and asking questions, which turned into published research along the way. The extremely applied work he carried out during and immediately after COVID-19 put his transition and post-physics research into application and context. There are, indeed, many ways to contribute and be a good scientist.

Today, both DH and DB are proud of doing their best to advance public health research related to infectious and chronic disease and have both enjoyed two-plus decades of work in public research. Surely nothing better can be said about a career transition.

## 3. Discussion

These ‘ten simple rules’ are not really rules. Instead, they are a set of suggestions for attitudes and perspectives that have helped the authors and other researchers to make transitions from basic science into public health research. They may provide useful starting points for researchers moving between other disciplines. For those with a research interest in career transitions themselves, future work could address correlates of successful career transitions with further qualitative and quantitative research. Success and fulfillment in a science career and in career transitions often go hand in hand with general perspectives and characteristics such as curiosity, making time to explore other disciplines, a passion for discovery, and a willingness to ask questions and listen to the answers [17]. Together, these traits help people to persist as lifelong learners—a clear requirement for a transition to a new research direction after your career has begun.
